# Factors associated with survival in patients with visceral leishmaniasis treated at a reference hospital in northern Minas Gerais - Brazil

**DOI:** 10.1590/0037-8682-0045-2024

**Published:** 2024-02-23

**Authors:** Igor Monteiro Lima Martins, Alfredo Maurício Batista de Paula, Antônio Prates Caldeira, Lanuza Borges Oliveira, Luciano Freitas Fernandes

**Affiliations:** 1 Universidades Estadual de Montes Claros, Programa de Pós-Graduação em Ciências da Saúde, Montes Claros, MG, Brasil.; 2 Universidade Estadual de Montes Claros, Departamento de Saúde da Mulher e da Criança, Montes Claros, MG, Brasil.; 3 Universidade Estadual de Montes Claros, Departamento de Enfermagem, Montes Claros, MG, Brasil.; 4 Universidade Estadual de Montes Claros, Hospital Universitário Clemente Faria, Montes Claros, MG, Brasil.; 5 Centro Universitário UNIFIPMOC, Montes Claros, MG, Brasil.

**Keywords:** Leishmaniasis, Visceral, Survival, Prognosis, Public Health

## Abstract

**Background:**

Visceral leishmaniasis (VL) is a public health problem and is a relevant cause of death in developing countries. This study aimed to evaluate the 20-year survival and predictors of worse prognosis in patients with VL admitted to a reference hospital for the treatment of infectious diseases between 1995 and 2016 in northern Minas Gerais, an area of high endemicity for VL.

**Methods:**

This retrospective cohort study was conducted at a hospital in northern Minas Gerais, Brazil. All patients with VL were evaluated over a 20-year period. The medical records were thoroughly analyzed. Cox regression analysis was performed to estimate factors associated with the probability of survival.

**Results:**

The cohort included 972 individuals, mostly male children <10 years old, from urban areas who presented at admission with the classic triad of fever, hepatosplenomegaly, and skin pallor. The mean hemoglobin level was 7.53 mg/dl. The mean interval between symptom onset and hospital admission was 40 days. The instituted therapies ranged from pentavalent antimonates to amphotericin, or both. The probability of survival was reduced to 78% one year after symptom onset. Hemoglobin levels and age were strongly associated with the probability of survival.

**Conclusions:**

Regardless of the mechanism underlying the reduction in hemoglobin and the non-modifiable factors of age, early initiation of drug treatment is the most appropriate strategy for increasing survival in patients with VL, which challenges health systems to reduce the interval between the onset of symptoms and hospital admission.

## INTRODUCTION

Visceral leishmaniasis (VL) is an infectious parasitic disease affecting neglected populations. VL is caused by the protozoa of the *Leishmania donovani* complex, which includes three species with typical morphological variations. For example, the *Leishmania donovani* species is the most common in the Old World (mainly in India), and in America, the *Leishmania (Leishmania) chagasi or Leishmania (Leishmania) infantum* ^1^ ^,^ ^2^ are more common. 

VL is the most severe form of leishmaniasis and causes a group of diseases with a broad clinical spectrum. These diseases are transmitted by infected female sand flies, i.e., it is a vector-borne disease. If not treated properly, VL is fatal in 95% of the cases^3^.

Clinically, VL commonly presents as prolonged fever, hepatosplenomegaly, and skin pallor. However, it can also result in weight loss and decreased appetite. From a laboratory point of view, pancytopenia is frequently detected with reduced hematimetric parameters, thus leading to anemia, leukopenia, and thrombocytopenia, which in turn favor the triggers coinfection and hemorrhagic events. Early diagnosis increases the likelihood of survival and is based on clinical, epidemiological, serological, and direct parasitological examination. Parasitological examination is the gold standard and is usually more expensive and more invasive^2^ ^-^ ^4^.

According to the World Health Organization (WHO)^3^, 90% of the cases reported in 2021 occurred in ten countries, Brazil, Ethiopia, Eritrea, India, Iraq, Kenya, Nepal, Somalia, South Sudan, and Sudan. 

In 20 years, Brazil has reported more than 50.000 cases of VL, and the WHO estimates that there are an average of 70.000 new cases of VL annually, suggesting that the cases could be underdiagnosed and underreported^5^. In Latin America, 70% of the cases occur in Brazil ^3^ ^,^ ^6^. Of the cases reported in Brazil, 43.5% occurred in children under 10 years of age, and the infection was most prevalent age group in the age group of 1-4 years (12.712 cases, 25.4%)^6^.

The Notifiable Diseases Information System (SINAN) showed an 85% increase in VL lethality from 1994 to 2004, increasing from 3.6% in 1994 to 6.7% in 2003, and to 8.4% in 2004^7^. Between 2007 and 2020, based on the number of confirmed cases and deaths from VL, the lethality rate was estimated to be 6.82%. This decrease could be explained by the introduction of new therapeutic options and intensified strategies to combat the vector and control urban reservoirs^6^ ^-^ ^10^. 

Regarding the survival of patients with VL, several factors have been identified as predictors of reduced survival probability and can be categorized into clinical, sociodemographic, laboratory, and contextual variables.

In the clinical variables: comorbidities^2^ ^,^ ^11^ ^-^ ^14^, hemorrhagic sites^2^ ^,^ ^11^ ^,^ ^13^ ^,^ ^15^, jaundice^2^ ^,^ ^11^ ^-^ ^12^ severe malnutrition^12^, long-term illness^12^, concomitant bacterial infections^14^ ^,^ ^15^, vomiting^11^, edema^11^, splenomegaly^11^ ^,^ ^12^, pneumonia^11^, bleeding on admission^16^, delay between onset of symptoms and admission more than 15 days^16^, waiting time between onset of symptoms and treatment^17^, and duration of treatment^17^. Jaundice, HIV coinfection, tuberculosis, edema, and bleeding were strongly associated with a negative prognosis in some studies (OR > 3)^2^ ^,^ ^11^ ^-^ ^17^. 

In the laboratory variables: elevated presence of some chemical mediators of immune response^2^, platelet count less than 50.000/mm3^11^, hemoglobin 6.5 mg/dl^12^, low total blood cell count, low platelets and high aspartate transaminase/alanine aminotransferase^14^, white blood cell count less than 4.000/mm3, and cytolysis^16^. 

In the sociodemographic factors, age^17^ presented as a heterogeneous aspect in the studies; specifically, age ≥60 years^12^ or <12 months^14^. Low education^18^ was also a significant predictor.

The contextual variables included lack of garbage collection, unemployment rate, low per capita income and income inequality^18^, district of residence^17^ high and low average temperatures, and road construction^13^.

Based on these covariates, the intervention measures can be more specific and given in a timely manner to optimize the disease prognosis.

This study aimed to evaluate the 20-year survival and predictors of worse prognosis in patients with VL admitted to a reference hospital for the treatment of infectious diseases between 1995 and 2016 in northern Minas Gerais, an area of high endemicity for VL.

## METHODS

### ● Ethical considerations

Although the present study used secondary data to analyze mortality in individuals with VL, this work was referred to the Ethics Committee on Human Research (CEP/Unimontes) and received a favorable opinion for its development (Opinion 1.471.595).

### ● Study Design

A retrospective cohort study was conducted in patients diagnosed with VL and followed up using medical records, medical charts, and an internal transfer control system. The follow-up time was 24 months in this study. The study was conducted at the Clemente de Faria University Hospital - HUCF (which is a reference in Leishmaniases), located in the city of Montes Claros, northern Minas Gerais - Brazil, 421.7 km from the capital Belo Horizonte and has an estimated population of 417.478 people^19^. The hospital is part of the administrative unit of Montes Claros State University UNIMONTES and has 151 beds. It is a public service funded by the state and federal resources^20^. 

### ● Data Collection

Data for up to 20 years prior to the study were collected. Data from January 1, 1995, to December 31, 2016 was collected. For data collection, an instrument was developed by the researchers themselves based on variables known to correlate with VL: demographic, socioeconomic, and laboratory variables; origin; diagnostic tests; selected therapy; and clinical evolution. 

All patients with VL hospitalized during the study period were investigated using a census. Data collection was carried out by professionals trained with the instrument terms and the data extracted from medical records. All data collection was supervised by a primary researcher.

Serological diagnosis was done using immunofluorescence and immunochromatographic methods or rapid tests, depending on availability. The tests followed the standardization of the Ezequiel Dias Foundation (FUNED), whose laboratory is a reference in the state of Minas Gerais (MG) for infectious and parasitic diseases, 

### ● Survival data

Specifically, for the assessment of survival, the follow-up time was determined from the appearance of the first signs and symptoms reported by the patient until failure. In this study, the failure event was death due to VL, and cases of hospital discharge, transfer to another service, and even death for other reasons were censored.

### ● Statistical Analysis

Statistical analyses were performed using the Statistical Package for the Social Sciences (SPSS version 24.0). Initially, the independent variables were presented descriptively, and then bivariate analyses were carried out using the simple Cox regression model at a significance level of p ≤ 0.2 to determine the variables to be included in the multiple model (COX regression). Next, the Kaplan-Meier method was used to estimate the probability of survival at each time point, with and without stratification.

Log-rank and Breslow tests were used to evaluate the survival curves of the strata of categorical variables, age and treatment, respectively.

Pearson's correlation test was used because the residuals of the variables that remained in the final model followed a normal distribution (age in years and hemoglobin), resulting in a null correlation between these covariates and follow-up time. The proportionality assumption for the COX regression model used to estimate the effect of covariates on survival was met. The final multiple model comprised of the variables which were statistically significant (p < 0.05).

## RESULTS

We identified 972 individuals (male to female ratio: 1.18:1; mean age: 12.9 ± 17.06 years) with confirmed VL diagnosis. Most individuals with VL were children from municipalities or districts in areas considered endemic for leishmaniasis (northern macro-regions and Jequitinhonha Valley of Minas Gerais State and southern macro-region of Bahia State). Approximately 20% of individuals resided in rural areas.

In more than 90% of the cases, these individuals presented with the classic triad of VL: fever, hepatosplenomegaly, and skin pallor. The diagnosis was often confirmed by serology, followed by clinical and/or parasitological tests. These individuals were treated with the standard first-line (e.g., pentavalent antimonate) and second-line medications, (e.g., amphotericin B), and in many cases (36.9%) both drugs were used for treatment when the treatment started with the first line but was replaced by the second line due to the adverse effects of the drug. 

However, it is important to note that, from 2011 to 2014, there was experience of treating patients with VL treated with both drugs simultaneously, according to a multicenter study that included the hospital unit of this study and later documented by Romero et al. in 2017.^21^. However, there was no difference to the standard treatment plan, which is first treatment with glucantime and then amphotericin, or in specific cases, amphotericin was the first choice for eligible patients. During hospitalization, the mean hemoglobin was 7.53 mg/dL (Table 1).

**TABLE 1:** Selected independent variables (demographic, clinical, biochemical and therapeutic) as predictors of survival probability in patients with visceral leishmaniasis admitted to HUCF between 1995 and 2016.

Deaths	Cases		Deaths		p*
	N	%	N	%	
**Gender**					600
Male	523	53,7	13	2,5	
Female	440	45,2	5	1,1	
**Age**					0,007
< 10 years old	640	65,7	5	0,8	
>10 years old	334	34,3	13	3,9	
**Source**					0,000
Montes Claros	386	39,6	4	1,0	
Other municipalities**	588	60,4	14	2,4	
**Residence**					0,270
Urban	792	81,6	13	1,6	
Rural	178	18,4	5	2,8	
**Fever**	954	98,8	17	1,8	0,000
**Hepatomegaly**	889	92,5	16	1,8	0,590
**Splenomegaly**	897	93,8	16	1,8	0,300
**Pallor**	915	95,5	17	1,9	0,006
**Diagnosis**					0,104
Clinical	79	8,3	2	2,5	
Serological	836	87,4	15	1,8	
Parasitological	42	4,4	1	2,4	
**Treatment**					0,000
Glucantime	251	26,1	1	0,4	
Amphotericin B	325	33,8	11	3,4	
Both	355	36,9	3	0,8	
Others	30	3,1	1	3,3	
Anemia***					0,830
Light	59	6,1	0	0,0	
Moderate	298	30,6	2	0,7	
Serious	577	59,2	16	2,8	
**Hemoglobin**					0,014
Mean	7,56		6,08		
Median	7,50		5,90		
Mode	6,50		5,10		
Minimum	2,20		3,40		
Maximum	13,50		8,30		

*Variables selected at the ≤ 20% level using simple cox regression model. **Municipalities in northern Minas Gerais, Jequitinhonha Valley, southern Bahia. ***Classification of the World Health Organization^40^.

The time interval between symptom onset and hospital admission with VL diagnosis was 39.8 days (± 54.81), ranging from two days to two years. The mean hospital stay was 18.41 days (± 9.86), ranging from one to ninety days. Among the patient who were followed-up, 18 patients (approximately 2% of the cases)n died. Of these, 72.2% were male, 27.8% were up to two years old, 72.2% lived in urban areas, Montes Claros was the place of origin (22.2%), and 61.1% were treated with amphotericin. A portion of the patients were transferred to other services (1.6%), and the majority were discharged during outpatient follow-up (96.5%). 

**FIGURE 1:**
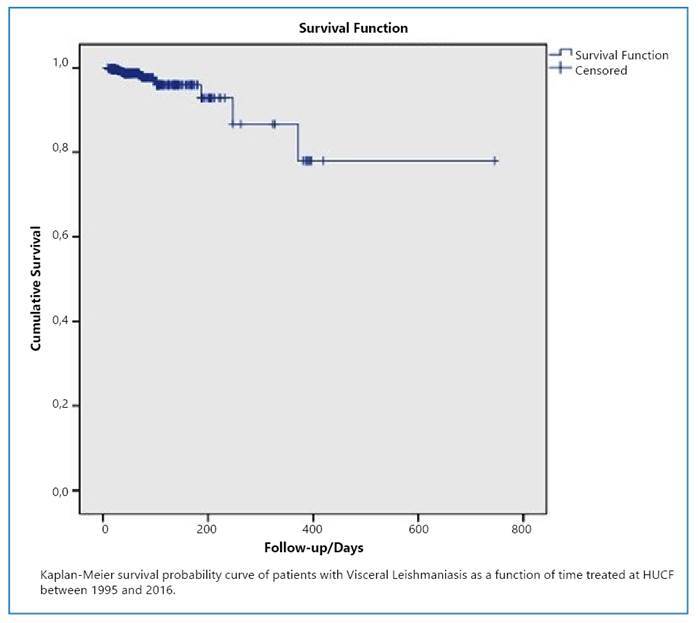
Survival function for Visceral Leishmaniasis among patients treated at HUCF between 1995 and 2016.

The Kaplan-Meier curve demonstrated that the probability of survival after one year of follow-up was 78% (Figure 1). The variable age in years was dichotomized into individuals <10 and >10 years to verify if there was a difference between the survival curves of younger and older individuals, but the log-rank test showed no statistical difference (probably due to sample fluctuation.

**FIGURE 2:**
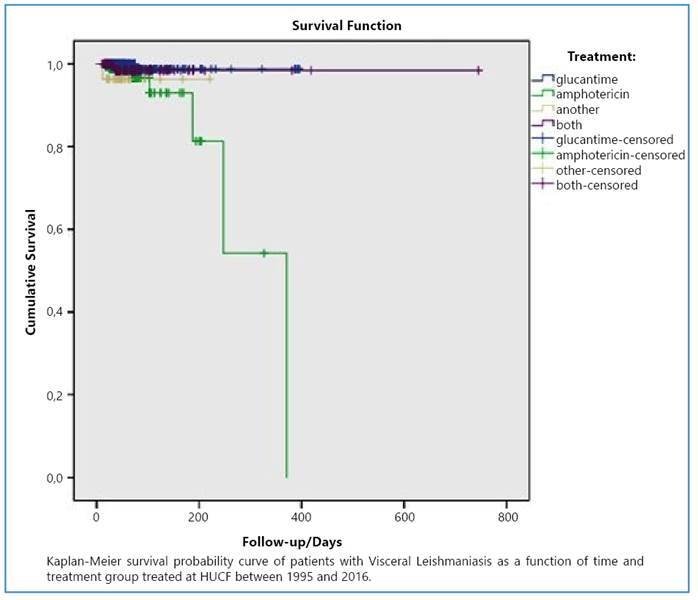
Survival of Visceral Leishmaniasis according to treatment among patients treated at HUCF between 1995 and 2016.

Another aspect highlighted was the difference in the therapeutic plans. The difference in survival between the treatments adopted based on the Breslow test was borderline (p = 0.054); however, it was not possible to rule out the possibility of differences in survival probability between treatments (Figure 2). 

The independent variables that remained in the final model were hemoglobin and age (Table 2). Using the multiple Cox regression model, it is possible to observe that an increase of 1 (one) unit in hemoglobin dosage reduced the hazard ratio for death by 44.7%, keeping the other variables (i.e. age) constant. Increasing patients life expectancy by one year increased the hazard ratio for death by 3%, keeping the other variables constant.

**TABLE 2:** COX regression model with proportional hazards for survival analysis of patients with visceral leishmaniasis seen at HUCF 1995 and 2016.

	B	P	HR	95% CI for HR	
				Lower	Upper
**Hb**	-0,592	0,000	0,553	0,407	0,751
**Age**	0,029	0,011	1,030	1,007	1,053

## DISCUSSION

This study of 972 individuals showed that the factors associated with survival in this study were age and hemoglobin 

Yeshaw et al^2^, in a retrospective cohort of 586 individuals showed that the factors associated with an increased incidence of mortality in patients with VL, included comorbidities, nosebleeds, treatment toxicity, jaundice, and being bedridden at admission. Based on the findings of the present study it can be suggested that nosebleeds might contribute to reduced hemoglobin and impact prognosis, as well as treatment toxicity, leading to a change in the therapeutic approach. 

Based on this, Costa et al^22^ had proposed bleeding and anemia as negative prognostic factors. The biological plausibility of bleeding and anemia in VL cases is correlated with increased levels of pro-inflammatory cytokines: IL-8, IL-1β, IL-10, and IFN-γ, which are also exacerbated in cases of bacterial coinfection. According to Moulik^23^, there is a positive correlation between the parasite load in VL and elevated levels of IL-10 in animal models. This suggests that this mechanism could be triggered by *Leishmania chagasi* for its survival in the body. However, the parasite-host interaction that sustains VL is still limited.

Another study^24^ corroborated these findings and demonstrated elevated levels of IL-10, IFN-γ, and TGF-β1 before the institution of treatment. The observed increase in serum IL-10 levels decreased remarkably within one week of treatment initiation. In addition, hematimetric parameters such as hemoglobin, leukocytes and platelets were restored to normal levels after the treatment was implemented. They findings further suggested the adoption of clinically associated IL-10 measurements as a criterion for curing the infection. 

It is important to highlight the work of Morimoto^25^ who demonstrated that in animal models he observed the hemophagocytic activity of macrophages infected with Leishmania parasites in animal models, the study showed the benefits of this procedure, that is, inducing the infected macrophage to perform phagocytosis of red blood cells for its survival

Thus, two possible mechanisms can explain the reduction of hemoglobin: bleeding favored by pro-inflammatory cytokines, and hemophagocytosis induced by the parasite.

Pinho et al^26^ observed a positive association between low expression of insulin-like growth factor-1 (IGF-1) and lower hemoglobin levels in patients with severe VL, suggesting a pathogenic mechanism different from that related to cytokine production and hemophagocytosis.

Henn et al^27^ investigated the laboratory characteristics of patients with VL coinfected with HIV, and measured the hematimetric parameters. That study showed lower levels of hemoglobin, lymphocyte count, and liver enzymes, and higher platelet and eosinophil counts in patients with VL coinfected with HIV when compared to patients with only VL. This increased the mortality outcome in those patients.

Mulaw et al^28^ proposed that iron and folic acid supplementation increased hemoglobin levels in patients with VL; however, this procedure favored the parasite. Parasite replication increases when trace elements are absorbed by amastigotes. The high affinity of protozoans for iron ions favors its survival and reproduction in intracellular environments.

The time between the onset of symptoms and treatment is a significant variable that determines the prognosis of the affected individuals^4^. Although this interval in this study was approximately 30 days on average, there were cases that remained for more than a year without proper diagnosis and necessary therapeutic intervention, suggesting a failure of the health system.

World Health Organization - OMS^3^ presented clear information that confirms the Kaplan-Meier curve of this study; a longer follow-up time indicates a significant reduction in the probability of survival. Therefore, early detection and development of drug therapies are important. 

This issue presents a challenge to health systems in developing countries. Training of health professionals, epidemiological surveillance, enhancement of human development, environmental management, and rational use of insecticides are strategies that can impact the health of neglected populations in relation to VL.

In this study, the difference in the Kaplan-Meier curve between the treatments adopted was borderline (p = 0.054), and it is not prudent to rule out the difference in the probability of survival between the therapeutic strategies or to consider them identical.

It is important to point out that there could have been a selection bias in this sample that influenced the Kaplan-Meier curve in terms of treatment-related survival. The most severely ill patients from the clinical and laboratory perspectives were selected for the amphotericin regimen, as recommended by the Brazilian Ministry of Health^8^. Thus, there was a greater propensity for death, which could have generated an association between this treatment and a lower chance of survival.

In a study by Yeshaw^2^ The drug toxicity in patients was shown to be a negative predictor of survival, with a death risk of 5.87 (95% CI 3.30-10.44) in relation to those who did not present adverse reaction. Anti-leishmania drugs in general have significant toxicity depending on the dose and condition of the individual. Arrhythmias and pancreatitis are possible manifestations in individuals undergoing anti-leishmania treatment^9^. 

Bulté^29^ described an increase in the infectivity of a strain of *Leishmania infantum* due to resistance to miltefosine, highlighting a possible therapeutic failure resulting from treatment of this parasite variant. Oliveira^30^ proposed the use of thiazolic derivatives as alternative treatments for VL. That in vitro study showed promising results, with lower toxicity to mammalian cells and evident leishmanicidal activity.

Currently, the recommended treatment regimen for patients with VL in Europe, the Americas, and the Mediterranean is liposomal amphotericin B at 20-21 mg/kg without HIV coinfection^31^ ^-^ ^34^. However, until the time of this study, as per the protocols of the Brazilian Ministry of Health, pentavalent antimoniate formulations was the first-line of treatment^9^. Cost is a limiting factor; however, through donations and public policies, liposomal amphotericin B may be adopted as a first option^8^ ^,^ ^9^ ^,^ ^35^. Other therapeutic strategies have been investigated in other studies^36^ ^,^ ^37^. 

The demographic profile of patients with VL is recurrent in the scientific literature: males, young (especially children under 10 years of age), living in urban areas^4^. Contrary to existing literature, in this study, increasing age (3%) was associated with an increased probability of death. 

The Pan-American Health Organization points out that VL affects extreme age groups such as children under five years and adults over 50 years (the most vulnerable groups)^38^. In a study by Carvalho et al^10^ the highest probability of death was observed in older age groups; which is consistent with the finidngs presented in another study^39^. In contrast, in the study by Salih et al^14^ the condition of an infant or child was associated with a higher probability of death (p = 0.02).

A study on premature mortality in patients with VL showed that children <5 years of age were diagnosed earlier than those in other age groups, and their survival was also lower than that in other age groups^35^. 

This discrepancy between findings can be explained by sample fluctuation and the confounding factor of age in relation to comorbidities, since the individual with advancing age are more likely to have comorbidities. In this study, the proportion of individuals in younger age groups was higher (more than 65% were less than 10 years old).

Thus, regardless of the underlying mechanism affecting the hemoglobin levels or age factor of patients with VL, early initiation of the recommended drug therapy is the best alternative for the survival in these individuals.

This study has several limitations. The study uses secondary data (such as medical records) that were not generated for research purposes, and because it is a teaching hospital, there are academic issues and turnover of staff members involved in care. Hence, the results should be considered with caution. Further, VL case notification forms were not used, which could increase the number of variables and produce a more robust analysis, although under-reporting is a problem, as it does not occur frequently in health services, and this becomes a point of attention in the context of this study^41^. However, the findings presented in this study can hep in future purposefully designed studies conducted to verify the impact of hemoglobin and age on the survival of patients with VL.
